# Characterization of promoter sequence of toll-like receptor genes in Vechur cattle

**DOI:** 10.14202/vetworld.2016.626-632

**Published:** 2016-06-21

**Authors:** R. Lakshmi, K. K. Jayavardhanan, T. V. Aravindakshan

**Affiliations:** 1 Department of Veterinary Biochemistry, College of Veterinary and Animal Sciences, Thrissur, Kerala, India; 2 Centre for Advanced Studies in Animal Genetics and Breeding, College of Veterinary and Animal Sciences, Thrissur, Kerala, India

**Keywords:** mastitis, promoter, sequence, toll-like receptor, Vechur breed

## Abstract

**Aim:**

To analyze the promoter sequence of toll-like receptor (TLR) genes in Vechur cattle, an indigenous breed of Kerala with the sequence of *Bos taurus* and access the differences that could be attributed to innate immune responses against bovine mastitis.

**Materials and Methods:**

Blood samples were collected from Jugular vein of Vechur cattle, maintained at Vechur cattle conservation center of Kerala Veterinary and Animal Sciences University, using an acid-citrate-dextrose anticoagulant. The genomic DNA was extracted, and polymerase chain reaction was carried out to amplify the promoter region of TLRs. The amplified product of TLR2, 4, and 9 promoter regions was sequenced by Sanger enzymatic DNA sequencing technique.

**Results:**

The sequence of promoter region of TLR2 of Vechur cattle with the *B. taurus* sequence present in GenBank showed 98% similarity and revealed variants for four sequence motifs. The sequence of the promoter region of TLR4 of Vechur cattle revealed 99% similarity with that of *B. taurus* sequence but not reveals significant variant in motifregions. However, two heterozygous loci were observed from the chromatogram. Promoter sequence of TLR9 gene also showed 99% similarity to *B. taurus* sequence and revealed variants for four sequence motifs.

**Conclusion:**

The results of this study indicate that significant variation in the promoter of TLR2 and 9 genes in Vechur cattle breed and may potentially link the influence the innate immunity response against mastitis diseases.

## Introduction

Toll-like receptors (TLRs) are critical sensors of microbial attack and effectors of the TLR dependent innate defense mechanism, enabling the host to eliminate pathogens that otherwise would cause disease or mortality [1]. TLRs recognize a wide variety of pathogen-associated molecular patterns (PAMPs) from bacteria, viruses, and fungi as well as some of the host molecules which in turn trigger intracellular signal transduction cascades that result in the expression of pro-inflammatory cytokines, chemokines, and antiviral molecules [2]. So far, 13 TLRs have been identified in mammals of which 10 TLRs are known to occur in cattle, and the expression of TLR transcripts varies among different mammalian species. Among the members of TLR family, TLR2, 4, and 9 play an essential role in both innate immunity and adaptive immune response by ligand recognition and signal transduction. TLR2 is essential for the recognition of a variety of PAMPs from Gram-positive bacteria, including bacterial lipoproteins, lipomannans, and lipoteichoic acids, whereas TLR4 is predominantly activated by lipopolysaccharides [3]. TLR9 is a pattern recognition receptor that plays a key role in cell survival through recognition of various bacterial components including unmethylated CpG-DNA [4]. The expression of TLR 2, 4, and 9 are critical sensors of innate defense against bacterial infection.

Vechur cattle, a rare breed of *Bos indicus*, are an indigenous breed of Kerala, and it is the smallest cattle breed in the world. They are well adapted for the hot, humid tropical climate of Kerala and are high disease resistant. In dairy industry, mastitis is considered to be one of the expensive diseases and a major economic issue for dairy farmers [5]. In India, the economic loss due to mastitis is about 2500 million per annum. Vechur breeds are not prone to mastitis. Characterization of factors involved in the innate immune system of this breed might provide an insight into the mechanisms involved in the disease resistance.

The promoter region of a gene, through binding of a specific transcription factor, is directly involved in gene transcription initiation. Therefore, sequence variation in this region may alter transcription factor binding sites, which in turn can affect gene expression and exert biological impacts [6]. Given their key role as sentinels of the innate immune defense, TLR structure, and function are tightly regulated. Numerous attempts have been made to identify variation affecting TLR structural genes, but studies on genetic variation in the promoter region of TLRs gene are relatively rare, and their contribution to disease remains unclear. In this study, promoter sequence of TLR2, 4, and 9 genes in Vechur cattle breed was examined and accessed the differences that could be attributed to innate immune responses against bovine mastitis.

## Materials and Methods

### Ethical approval

The study was approved by the committee framed for the research by the university authority. Adequate measures were taken to minimize pain or discomfort in accordance with the International Animal Ethics Committee.

### Experimental animals and DNA extraction

Blood samples were collected using acid-citrate-dextrose anticoagulant from Jugular vein of Vechur cattle, maintained at Vechur cattle conservation center of Kerala Veterinary and Animal Sciences University. The genomic DNA was extracted by phenol-chloroform method. The DNA concentration was assessed by NanoDrop (Thermo Scientific, USA) spectrophotometer, and the purity was confirmed by measuring absorbance at 260 nm and 280 nm, followed by quality of the DNA was also assessed by agarose gel (1%) electrophoresis.

### Primer designing and polymerase chain reaction (PCR) amplifications

Primers used to amplify the promoter region of TLR2 (AC_000174), TLR4 (AC_000165), and TLR9 (AC_000179) genes in Vechur cattle were designed from *Bos taurus* sequences available in National Centre for Biotechnology Information (NCBI) database. Primers were designed using an online tool from NCBI, Primer-BLAST. The designed forward and reverse primers were custom synthesized at Sigma-Aldrich India (Table-1). The primers were reconstituted in nuclease-free water to a concentration of 10 p M/µl.

**Table-1:** Primers designed for TLR promoter regions from NCBI databank sequences.

Target	Primer sequence (5’→3’)		Size
Promoter region of TLR2	Forward	TGTGGCATCTCTCGTTTCCT	934 bp
	Reverse	CTGGTTACTCTGCTCCCTGA	
Promoter region of TLR4	Forward	GTCCCTTGCTCTATCAGGCA	898 bp
	Reverse	ATGCTGTCCCCTTGGCTTAT	
Promoter region of TLR9	Forward	CTGGGGTAGGGGCTTTATAAGA	1005 bp
	Reverse	CCATCTGTCACATCCCACGT	

TLR=Toll-like receptor

The promoter regions of TLRs were amplified through PCR. 20 µL of PCR were carried out in 0.2 mL PCR tube and each tube contained 2 µL of genomic DNA (50 ng) as template, 1µl of each forward primer and reverse primer (10 pM/µl), ×2 concentration of 10 µl of PCR reaction mix (contain dNTPs- 0.4 mM each, Taq polymerase-0.05 U/µl, magnesium chloride-4 mM), and 6 µL of nuclease-free water. PCR conditions followed for amplification of promoter region of TLR2, 4, and 9 genes are presented in Table-2. Amplified PCR products were separated by agarose gel electrophoresis (1.5%) and visualized by ethidium bromide staining.

**Table-2:** PCR program for amplification of TLR promoter region in Vechur cattle.

Steps	Temperature and time
Initial denaturation	95°C for 3 min
Denaturation	95°C for 30 s
Annealing	53.2°C for 30 s for TLR2 56.0°C for 30 s for TLR4 53.0°C for 30 s for TLR9
Extension	72°C for 1 min Step 2 to 4 set for 35 cycles
Final extension	72°C for 5 min

TLR=Toll-like receptor, PCR=Polymerase chain reaction

### Sequencing and analysis

The purified PCR products of the promoter regions of TLR2, 4, and 9 genes were commercially sequenced by Sanger’s enzymatic DNA sequencing technique. The nucleotide sequences for the promoter of TLR2, 4, and 9 genes were submitted to GenBank. Sequence alignment of Vechur breed promoter regions of TLR2, 4, and 9 genes with *B. taurus* sequences was performed using CLUSTALW.

## Results and Discussion

Bovine mastitis, defined as an inflammation of the mammary gland, is generally considered the most economically imposing diseases of dairy cattle. Financial losses due to mastitis occur for animals experiencing both subclinical and clinical disease. Subclinical mastitis is the most economically important form of mastitis because of long-term reductions in milk yield. Vechur cattle breed found in Kerala state are known for less susceptible to mastitis than any other cattle breeds. The marked differences in susceptibility to mastitis predict that there is substantial variation in the efficiency of the antimicrobial defense within the cattle breeds. Yet, the mechanisms underlying exaggerated or attenuated response of Vechur breed to mastitis remain unclear. As TLRs are essential for innate immunity, understanding the genetic basis of varied TLRs receptor expression and function is of great importance for the many biological end points that depend on TLRs signaling. The promoter region of TLRs plays a key role in the transcription of genes. Promoter region consists of various consensus sequences, capable of regulating the rate of transcription by inducing or suppressing the respective genes. TLR2, 4, and 9 are reported as critical sensors of innate defense against bovine mastitis [3]. In this study, sequence variation of these TLRs promoter region in Vechur breed was examined for the important regulatory motifs that might influence the expression of TLR genes and innate immune response dynamics against bovine mastitis.

**Figure-1:**
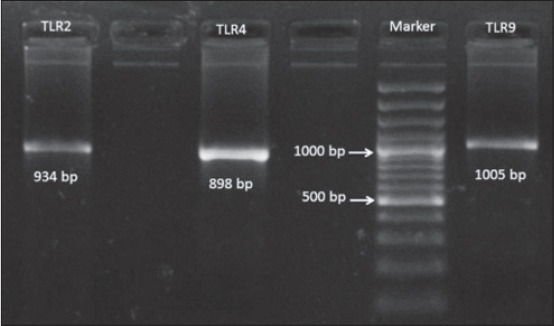
Polymerase chain reaction product size of toll-like receptor 2, 4, and 9 promoter region.

The promoter region of TLR2, 4, and 9 of Vechur cattle was successfully amplified using specific primer pairs. The sizes of amplified products were verified using agarose gel electrophoresis (Figure-1). Nucleotide sequences for promoter regions of these TLRs genes were deposited in GenBank and assigned with the following accession numbers: TLR2 promoter, KR559022.1; TLR4 promoter, KR559023.1; TLR4 promoter, KR559024.1.

**Figure-2:**
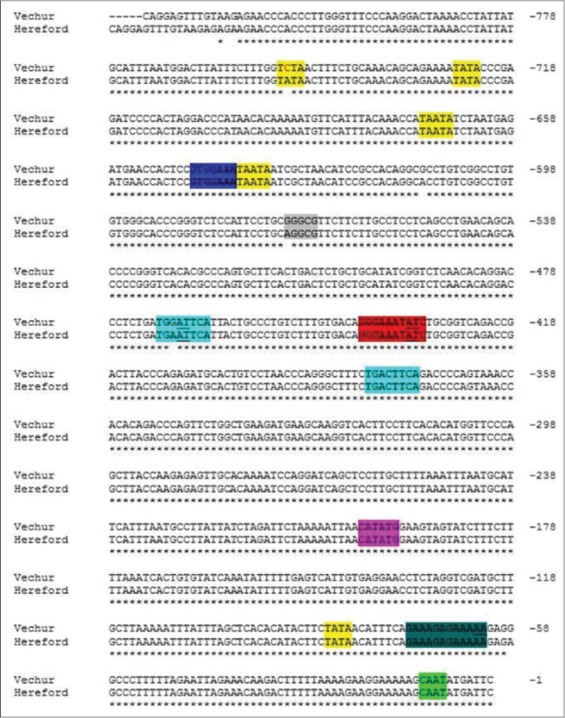
Sequence alignment of toll-like receptor 2 promoter region of Vechur cattle breed and Bos taurus (Hereford) highlighted with important motifs.

TLR2 is essential for the recognition of a variety of PAMPs from Gram-positive bacteria. TLR2 forms heterodimers with TLR1 or TLR6, each dimer having different ligand specificity thus increasing its binding receptors. TLR2 mRNA expression was strongly increased in mammary tissue in cattle. The promoter sequences of TLRs genes in Vechur cattle were assessed with *B. taurus* for the important sequence motifs, consensus sequence, and their functions. The sequence of promoter region of TLR2 of Vechur cattle with the *B. taurus* sequence present in GenBank showed 98% similarity and revealed variants for four sequence motifs. All of these motifs were located in sequences with a high degree of homology to possible transcription factor binding site. The important sequence motif and variations observed in Vechur cattle with that of *B. taurus* sequence are listed in Table-3 and also highlighted in Figure-2.

**Table-3:** Important sequence motifs and variants observed in the TLR2 promoter region of *B. taurus* and Vechur cattle breed.

Motif	Consensus sequence	Region	*B.taurus* sequence	Vechur sequence
TATA sequence	TATA	−726 to−723 −85 to 81 −752 to−748	TATA TATAA TATAA	TATA TATAA T**C**TAA
CRE	TGACGTCA	−379 to−372 −470 to−463	TGAC**T**TCA TGA**AT**TCA	TGAC**T**TCA TG**GAT**TCA
E-box	CANNTTG	−200 to−195	CATATG	CATATG
EC	GTGG (A/T)(A/T)(A/T)	−645 to−639	GTGGAAA	GTGGAAA
CAAT	CAAT	−11 to−8	CAAT	CAAT
NF-kB	GGGRNNYYCC, R-purine, Y-pyrimidine	440 to 431	GGGAAAT**AT**C	GGGAAAT**AT**C
IRF	GAAANNGAAAGG	−73 to−62	GAAAGAGAAA**AA**	GAAAGAGAAA**AA**
Sp-1 site	GGGCGG	−571 to−567	**A**GGCG	**G**GGCG

EC=Enhancer core, TLR=Toll-like receptor, CRE=Cyclic adenosine monophosphate responsive elements, NF-kB=Nuclear factor-kappa B, IRF=Interferon regulatory factor 3, *B. taurus*=*Bos taurus*

A consensus TATA boxwas observed in the promoter sequence of TLR2 gene of Vechur, which might consider as the core promoter for transcription initiation [7], to which RNA polymerase II binds is found to be present in the region between −726 and −723 bp. In addition, TATA-like sequences such as TATAA also present in the region of −85 to −81 bp and −752 to −748 bp with one single nucleotide polymorphism (SNP) at bp −751 in Vechur cattle. The AT-rich region of TATA box can facilitate easy unwinding of DNA due to weak interaction between the bases than GC during initiation of transcription.

Cyclic adenosine monophosphate (cAMP) responsive elements (CRE) are ubiquitous regulator of inflammatory and immunological reactions [8]. CRE is expressed in a wide variety of cell types. It has been established that cAMP induces phosphorylation of CRE, which then activates cAMP-responsive genes, leading to increased cell proliferation, differentiation, or modulation of various cell functions [9]. TLR2 found to be mediated by cAMP production [8]. CRE-like sequence (TGACGTCA) is detected at two positions in both *B. taurus* and Vechur sequence. First, CRE is positioned at −379 to −372 bp with one SNP at bp −375 in both Vechur cattle and *B. taurus*, and second, CRE is positioned at −470 to −463 bp is observed with three mismatch base pair in Vechur and two mismatch base pair in *B. taurus*.

Nuclear factor-kappa B (NF-kB) transcription factor was observed at position −440 to −431 bp with two mismatch base pairs in both Vechur and *B. taurus* sequence with reference consensus sequence. NF-kB consists of a family of transcription factor that play critical roles in inflammation, immunity, cell proliferation, differentiation, and survival [10]. This NF-kB plays an important role in the regulation of TLR2 gene expression [11]. The special protein binding sites (Sp site), which enhance the expression of TLR2 gene [12] is also present in the region between −571 and −567 bp in Vechur, whereas *B. taurus* reveals one mismatch at −571 bp.

**Figure-3:**
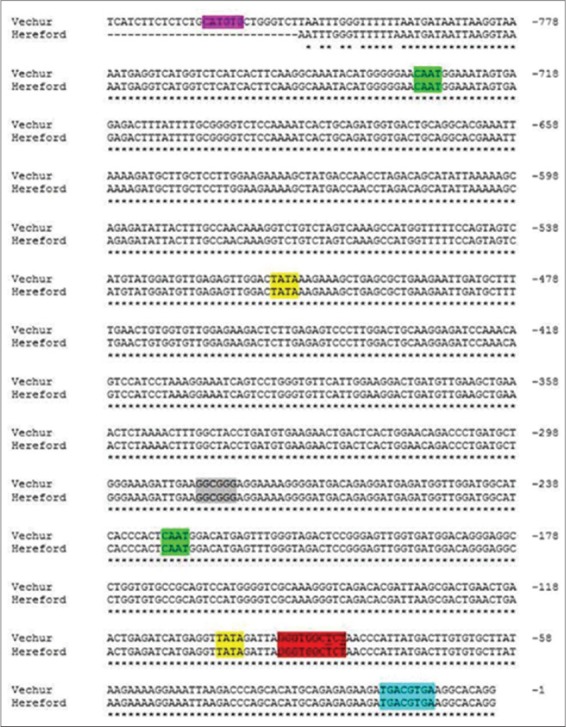
Sequence alignment of toll-like receptor 4 promoter region of Vechur cattle breed and Bos taurus (Hereford) highlighted with important motifs.

**Figure-4:**
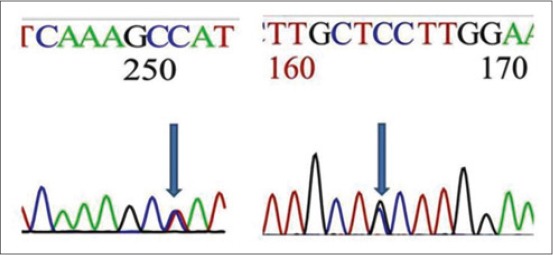
Sequence chromatographs reveal heterozygous peaks in the promoter region of toll-like receptor 4 gene.

In the present study, the sequence of the promoter region of TLR4 of Vechur cattle revealed 99% similarity with that of *B. taurus* sequence and had important motifs such as TATA, CAAT, E-box, NF-kB, CRE, and CpG regions required for regulation of transcription but not reveals significant variant in motif regions. The important sequence motif for TLR4 observed in Vechur cattle with that of *B. taurus* sequence are listed in Table-4 and highlighted in Figure-3. TLR4 plays an important role in the induction of the inflammatory response by recognizing exogenous PAMPs and endogenous ligands [13]. TLR4 is linked to the activation of NF-κB factor in several cell types [14]. Increased NF-κB activity was found in the milk and intra-mammary epithelial cells of mastitis-affected cows. Although Vechur promoter sequence of TLR4 did not show any polymorphism with *B. taurus* sequence, however, chromatograph reveals two heterozygous conditions in Vechur breed (Figure-4).

**Table-4:** Important sequence motifs and variants observed in the TLR4 promoter region of *B. taurus* and Vechur cattle breed.

Motif	Consensus sequence	Region	*Bos taurus* sequence	Vechur sequence
TATA	TATA	−513 to−510 −101 to−98	TATA	TATA
E-box	CANNTTG	−823 to−818	CATGTG	CATGTG
CRE	TGACGTCA	−17 to−10	TGACGTGA	TGACGTGA
CAAT	CAAT	−732 to−729 −229 to−226	CAAT	CAAT
NF-kB	GGGRNNYYCC, R-purine, Y-pyrimidine	−92 to−81	GGGTGGCTCT	GGGTGGCTCT
Sp-1 site	GGGCGG	−284 to−278	GGGCGG	GGGCGG

EC=Enhancer core, TLR=Toll-like receptor, CRE=Cyclic adenosine monophosphate responsive elements, NF-kB=Nuclear factor-kappa B, *B. taurus=Bos taurus*

**Figure-5:**
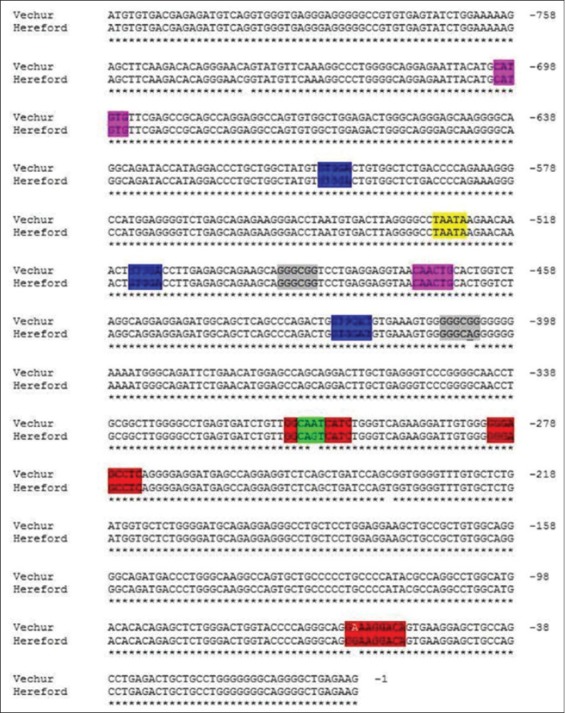
Sequence alignment of toll-like receptor 9 promoter region of Vechur cattle breed and Bos taurus (Hereford) highlighted with important motifs.

Promoter sequence of TLR9 gene also showed 99% similarity to *B. taurus* sequence and revealed variants for four sequence motifs. The sequence variation in TLR9 promoter region for the important motifis presented in Table-5 and Figure-5. TLR9, which is localized intracellularly, is involved in the recognition of specific unmethylated CpG-oligodeoxynucleotides (ODN) sequences that distinguish bacterial DNA from mammalian DNA. Bacterial DNA can stimulate immune cells mainly because of the unmethylated CpG motifs, which are rarely detected in vertebrate DNA, and if present, are highly methylated. Species difference in TLR9 expression during mastitis exists as CpG-ODN has been shown to promote the expression of its specific receptor (TLR9 mRNA) in goat mammary tissue [15].

**Table-5:** Important sequence motifs and variants observed in the TLR9 promoter region of *B. taurus* and Vechur cattle breed.

Motif	Consensus sequence	Region	*B. taurus* sequence	Vechur sequence
TATA sequence	TATA	−529 to−525	T**A**ATA	T**A**ATA
E-box	CANNTTG	−700 to−695 −72 to−467	CATGTG CAACTG	CATGTG CAACTG
EC	GTGG (A/T) (A/T) (A/T)	−606 to−602 −514 to−510 −424 to−419	GTGGA GTGGA GTGGAT	GTGGA GTGGA GTGGAT
CAAT	CAAT	−309 to−306	CA**G**T	CAAT
NF-kB	GGGRNNYYCC, R-purine, Y-pyrimidine	−281 to−271 −311 to−302 −62 to−54	GGGAGCCTC GGCA**G**TCATC GGAAGGACA	GGGAGCCTC GGCA**A**TCATC G**A**AAGGACA
Sp-1 site	GGGCGG	−492 to−487 −408 to−402	GGGCGG GGGGC**A**G	GGGCGG GGGGCGG

EC=Enhancer core, TLR=Toll-like receptor, NF-kB=Nuclear factor-kappa B, *B. taurus=Bos taurus*

The promoter sequence of TLR9 in Vechur and *B. taurus* shows variation for enhancer core (EC) region, CAAT box, nuclear factor-kappa binding protein, and Sp-1 binding site. EC region is expressed in a variety of tissues, and transgenic animal studies have demonstrated that EC region plays a critical role in thymocyte and macrophage development [16]. Three EC regions were observed in the sequence of Vechur cattle. CAAT box region also found in Vechur, however, *B. taurus* sequence reveals variation for this box at −307 bp. ECs, CAAT enhancer binding protein might physically and functionally interact with each other, leading to maximal transcription of the TLR9 gene [17].

NF-kB was observed at three positions; one SNP was observed in both Vechur and *B. taurus* at −307 bp, respectively. Vechur sequence also reveals another SNP in the NF-kB region of −61 bp. Sp1 binding sites were noticed at two regions, no variation was observed between Vechur and consensus sequence for this binding sites, however, *B. taurus* reveals an SNP at −403 bp.

## Conclusion

The TLR2 and TLR9 promoter regions are considerably more variable than TLR4 in Vechur breed to that of *B. taurus* as revealed by four different important motifs which further identified with SNPs. TATA and CAT boxes and multiple putative binding sites present in the TLR2 and TLR9 promoter sequences may influence the transcription. This study is, therefore, the first to propose that genetic variation in the TLRs promoter might influence the TLRs expression. A significant finding is the identification of SNPs in the promoter of TLR2 and 9 genes in Vechur cattle breed. The variation in TLR promoter sequence of Vechur breed might potentially influence the innate immunity response against mastitis.

## Authors’ Contributions

RL - The research work mentioned in this article is a part of the Ph.D. research work of the first author and first author carried out all the work mentioned in this article. KKJ - The entire work mentioned in this article was carried out under the guidance and supervision of the second author. TVA- Member of research advisory committee reviewed the manuscript. All authors read and approved the final manuscript.
